# Evaluation of the Prevalence and Production of *Escherichia coli* Common Pilus among Avian Pathogenic *E. coli* and Its Role in Virulence

**DOI:** 10.1371/journal.pone.0086565

**Published:** 2014-01-23

**Authors:** Alyssa K. Stacy, Natalie M. Mitchell, Jacob T. Maddux, Miguel A. De la Cruz, Laura Durán, Jorge A. Girón, Roy Curtiss 3rd, Melha Mellata

**Affiliations:** 1 The Biodesign Institute, Arizona State University, Tempe, Arizona, United States of America; 2 School of Life Sciences, Arizona State University, Tempe, Arizona, United States of America; 3 University of Florida, Gainesville, Florida, United States of America; Cornell University, United States of America

## Abstract

Avian pathogenic *Escherichia coli* (APEC) strains cause systemic and localized infections in poultry, jointly termed colibacillosis. Avian colibacillosis is responsible for significant economic losses to the poultry industry due to disease treatment, decrease in growth rate and egg production, and mortality. APEC are also considered a potential zoonotic risk for humans. Fully elucidating the virulence and zoonotic potential of APEC is key for designing successful strategies against their infections and their transmission. Herein, we investigated the prevalence of a newly discovered *E. coli* common pilus (ECP) for the subunit protein of the ECP pilus (*ecpA*) and ECP expression amongst APEC strains as well as the role of ECP in virulence. A PCR-based *ecpA* survey of a collection of 167 APEC strains has shown that 76% (127/167) were *ecpA+*. An immunofluorescence assay using anti-EcpA antibodies, revealed that among the *ecpA*+ strains, 37.8% (48/127) expressed ECP when grown in DMEM +0.5% Mannose in contact with HeLa cells at 37°C and/or in biofilm at 28°C; 35.4% (17/48) expressed ECP in both conditions and 64.6% (31/48) expressed ECP in biofilm only. We determined that the *ecp* operon in the APEC strain χ7122 (*ecpA+*, ECP-) was not truncated; the failure to detect ECP in some strains possessing non-truncated *ecp* genes might be attributed to differential regulatory mechanisms between strains that respond to specific environmental signals. To evaluate the role of ECP in the virulence of APEC, we generated *ecpA* and/or *ecpD*-deficient mutants from the strain χ7503 (*ecpA+*, ECP+). Deletion of *ecpA* and/or *ecpD* abolished ECP synthesis and expression, and reduced biofilm formation and motility *in vitro* and virulence *in vivo*. All together our data show that *ecpA* is highly prevalent among APEC isolates and its expression could be differentially regulated in these strains, and that ECP plays a role in the virulence of APEC.

## Introduction

Avian Pathogenic *E. coli* (APEC), a subgroup of Extraintestinal Pathogenic *E. coli* (ExPEC), is the etiologic agent of colibacillosis in birds. Colibacillosis, responsible for significant economic losses in the poultry industry worldwide, includes multiple extra-intestinal diseases often respiratory, leading to systemic or localized infections depending on the strain, age and the gender of the host, as well the immunologic status and the presence of predisposing environmental conditions [1], [2].

Multiple virulence factors are associated with APEC and are determined to be involved in different steps of their infection and/or fitness, including colonization (Type 1, P, AC/1, Stg fimbriae, type IV pili, curli, Tsh), invasion (IbeA, Tia), iron acquisition (aerobactin, salmochelin, SitABCD, a heme utilization/transport protein ChuA), serum-complement resistance (TraT, Iss, LPS, K1 capsule), antiphagocytic activity (O and K antigens, SitABCD), and virulence genes regulation (BarA-UvrY, Pts). At different steps of infection, ExPEC, including APEC could use alternative virulence factors. The nature and the combination of virulence factors associated with ExPEC could determine the degree of their virulence and their potential to cause specific diseases in specific hosts.

APEC share important virulence traits with human ExPEC, including uropathogenic *E. coli* (UPEC) and neonatal meningitis *E. coli* (NMEC), which render them a possible zoonotic risk or a reservoir of virulence genes for human strains [3].

*E. coli* common pilus (ECP), originally named Mat (meningitis-associated and temperature-regulated), was first identified in neonatal meningitis *E. coli* (NMEC) isolates [4] and later in all classes of pathogenic and non-pathogenic *E. coli* [5]. ECP, considered as a new variant of the chaperon-usher (CU) fimbriae family, is composed of a polymerized EcpD tip adhesin and a major shaft major pilin EcpA [6], and is encoded by the operon *ecpRABCDE* [5].

*In vitro* studies have shown that ECP plays a dual role in early-stage bacterial biofilm formation and host cell recognition in human pathogenic *E. coli* [7]–[9]. The purpose of this study is to evaluate the prevalence of *ecp* among APEC, its expression under two *in vitro* conditions and to determine its role in virulence in baby chicks. We present the first study on the role of ECP in a non-human pathogenic *E. coli*, APEC. Our data revealed new insights into ECP expression in *E. coli* and determined for the first time the role of ECP *in vivo* and in multiple virulence-associated phenotypes in APEC.

## Results and Discussion

### *ecpA* is Highly Prevalent among APEC Isolates

ECP, first detected in NMEC isolates [4], was found to be common among pathogenic and non-pathogenic *E. coli* [5]. Recent studies have determined that *ecpA,* the gene of the major pilin of ECP, was prevalent among the majority of human pathogenic *E. coli*; it was in fact shown to be highly associated with atypical enteropathogenic *E. coli* (aEPEC) (86%) [10], enteroaggregative *E. coli* (EAEC) (96%) [7], and enterotoxigenic *E. coli* (ETEC) (80%) [11] isolates. In our previous study, we detected the presence of *ecp* in a few APEC strains tested along with other human pathogenic *E. coli* [5], but there are no studies on the prevalence of *ecp* among animal pathogenic *E. coli*, including APEC. Herein, for the first time, we assessed the prevalence of *ecp* among APEC isolates. A PCR-based *ecpA* survey performed on a collection of 167 strains of which 166 clinical isolates were from diseased chickens and turkeys with signs of colibacillosis [12] [This study], and one APEC reference strain χ7122 (O78:K80:H9) [13], has determined that, similar to human enteric and septicemic *E. coli* isolates, the vast majority (76%; 127/167) of APEC isolates possess the *ecpA* gene. These data confirm that APEC share virulence genes with human pathogenic *E. coli,* and this gene which is common among intestinal and extra-intestinal pathogenic *E. coli* could be involved in the persistence of these bacteria in some environments, such as intestines, where they have a commensal life-style before causing diseases in different sites.

### APEC Strains Express ECP Differently in Biofilm and in Contact with HeLa Cells

**Figure 1:**
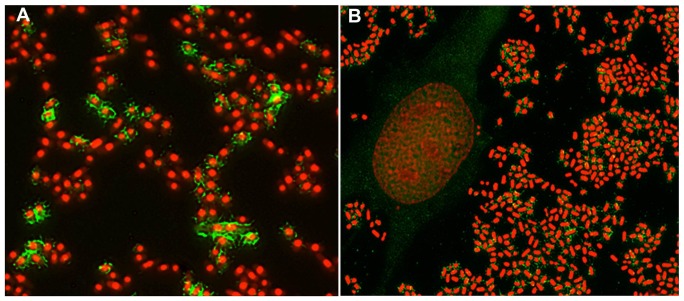
Demonstration of ECP expression on a representative APEC strain χ7234. The presence of ECP (green) on the bacteria (red) associated biofilm (**A**), or adhering to HeLa cells (**B**) was demonstrated using an anti-EcpA antibody (dilution 1∶2,000). Bacteria were previously incubated overnight at room temperature in DMEM with 0.5% mannose before the 6-H infection of HeLa cells. Biofilms were allowed to form 48 h at 28°C in DMEM with 0.5% mannose.

**Figure 2:**
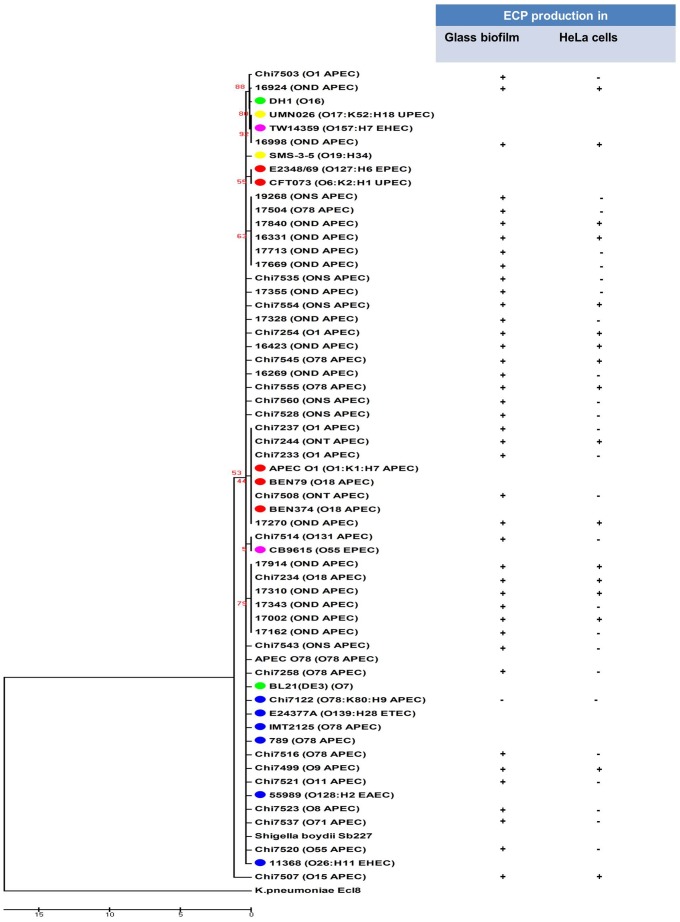
Neighbor-joining phylogenetic tree from analysis of *ecp* upstream region of APEC strains. Phylogenetic relationships between nucleotide sequences of putative *ecp* promoter sequences (−603 bp to −1 bp from the start codon GTG of *ecpR*) of APEC strains sequenced in this study (n = 40) or those available on public database (n = 7) [APEC-O1 (NC_008563.1); BEN374 (JN377377); BEN79 (JN377376); 789 (JN377380), APEC-O78 (NC_020163.1), χ7122 (NZ_HE962388.1), IMT2125 (NZ_HE964769)]. Eleven related sequences of human pathogenic and non-pathogenic *E. coli* [(DH1 (CP001637), UMN026 (CU928163.1), TW14359 (CP001368), SMS-3-5 (CP000970), CB9615 (CP001846), CFT073 (NC_004431.1), E2348/69 (FM180568.1), E24377A (CP000800), BL21(DE3) (NC_012892.2), 55989 (CU928145.2), 11368 (AP010953)] and one sequence of *Shigella boydii* Sb227 (NR_076357.1) were included as controls and the sequence of *Klebsiella pneumoniae* Ec18 (NZ_HF536482.1) was used as outgroup. Results of ECP expression of APEC strains tested in biofilm or in contact with HeLa cells are shown on the right. Colored dots represent phylogenetic groups, Green (A), Blue (B1), Red (B2), Yellow (D), and Purple (E). Abbreviations: NS, non-specific; NT, non-typable; ND, not determined.

Previous studies have shown that ECP expression in both diarrhoeagenic and meningitic *E. coli* is under the control of environmental cues [4], [5], [14]. Environmental conditions that up-regulate ECP expression include low pH, high acetate concentration [14] and low growth temperature in NMEC [4] and DMEM with 5% CO_2_ in diarrhoeagenic *E. coli* [5]. In our study, evaluation of ECP expression in *ecpA*+ APEC strains grown in DMEM +0.5% Mannose in both biofilm at 28°C and in contact with HeLa cells at 37°C has shown that strains behaved differently in their ECP expression. The immunofluorescence assay using anti-EcpA antibodies revealed that among the *ecpA*+ strains, 37.8% (48/127) expressed ECP when grown in DMEM +0.5% Mannose, both in contact with HeLa cells and/or in biofilm (Fig. 1 and 2); 35.4% (17/48) expressed ECP in both conditions and 64.6% (31/48) expressed ECP in biofilm only.

We suspected that the inability of some APEC strains to express ECP could be due to a truncation in their *ecp* genes. However, the analysis of the DNA of the *ecp* operon in the genome of the prototype APEC strain χ7122 (O78:K80:H9), which tested *ecpA*+ and ECP- (Fig. 2), has determined that the *ecp* operon in χ7122 was not truncated (Fig. S1). Comparison of the χ7122 *ecp* operon with those of two fully sequenced human strains, UPEC CFT073 and ETEC E2348/69, which *ecp* operons were determined to be functional [6], [14], has confirmed the organization of its homologous genes *ecpRABCDE* [6], [8] in the genome of χ7122 (Fig. S1) and was similar to those of the two strains CFT073 (NC_004431.1) and E2348/69 (FM180568.1). The identities of the proteins encoded by the *ecp* operon were between 96%–99% similar (Table S1). The failure to detect ECP in some strains possessing *ecp* genes, including APEC χ7122, might be attributed to differential regulatory mechanisms between strains that respond to specific environmental signals [5]. We thus proceed with more analysis on the O-group and the *ecp* upstream region in the strains, as described below.

### ECP Expression in APEC in the Conditions of this Study is not Serologically or Phylogenetically Group-associated

The first report on ECP expression in *E. coli* has determined that NMEC expressing ECP at 20°C were from the same serogroup O18:K1:H7 [4]. Herein, APEC isolates tested belong to different O-antigen groups (Fig. 2) and the expression of ECP in these APEC strains does not correlate with their O-antigen type. As a matter of fact, APEC that expressed ECP in the conditions tested were from different serogroups, including O1, O78, O9, O15, O18, O131, O55, O11, O8, O45, and O71 (Fig. 2). Moreover, strains from the same serogroup behaved differently in their ECP expression. For example, among seven O1 isolates tested for ECP expression, as described in material and methods, 3 were ECP-negative, 3 expressed ECP in biofilm only, and 1 expressed ECP in both biofilm and in contact with HeLa cells. Among the 18 O78 isolates tested, 13 did not express ECP, 3 expressed ECP in biofilm only, and 2 expressed ECP in both biofilm and in contact with HeLa cells (Fig. 2).

We next analyzed and compared the upstream *ecp* operon region of APEC isolates tested *ecpA^+^* and ECP^+^. A study by Lehti *et al*. [15] has correlated ECP expression in *E. coli* with phylogenetic group-associated promoter lineages and according to their analysis, strains from the B2/D/E lineage groups grown in the host environmental conditions (low pH and high acetate concentration) expressed ECP, whereas strains from lineage A/B1 did not. Herein, some APEC strains grown in biofilm or in contact with HeLa cells expressed ECP either in both conditions or in biofilm only. To determine if the difference in ECP expression is related to the heterogeneity of their *ecp* promoter, we compared the nucleotide sequence in the upstream region of *ecpR* (−603 bp to −1 bp) in multiple APEC strains from which the sequences were available or sequenced in this study (Fig. 2). The phylogenetic tree generated confirmed that the operon region of *ecpR* in APEC strains are heterologous and are regrouped in different distinct clusters [14] (Fig. 2); but contrary to the study by Lehti *et al*. [15], the expression of ECP in APEC strains in our conditions does not correlate with the *ecp* upstream DNA sequence. However, the ECP expression in the conditions of this study was not phylogenetically group-associated.

### APEC Strain χ7234 Opsonized with Anti-ECP Antibodies was Deficient in Adherence to Epithelial Cells

**Figure 3:**
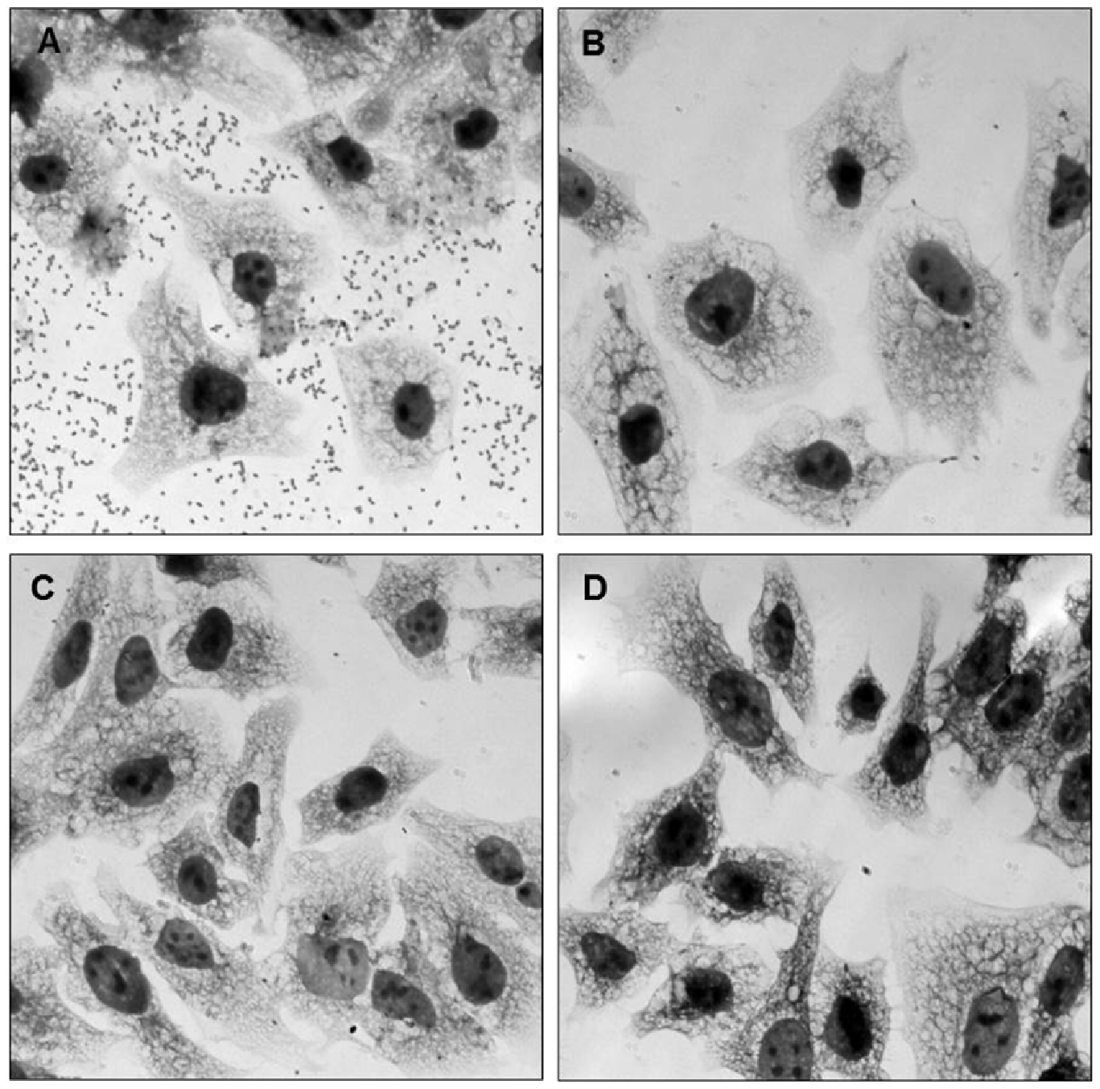
Inhibition of adherence by anti-ECP antibodies. Giemsa staining showing inhibition of APEC χ7234 adherence to HeLa cells with anti-ECP antibodies. **A**, No antibody; **B**, 1∶100; **C**, 1∶50; **D**, 1∶10.

The first step of bacterial infection is host-pathogen recognition. The tropism of bacteria is determined by the nature of their fimbriae/adhesins and along with other virulence factors, they cause specific diseases. APEC and human ExPEC share virulence factors and some APEC strains have the potential to cause human ExPEC diseases, especially urinary tract infection [16]. In this study, similar to human pathogenic *E. coli* [5], [7], [9], [17], APEC grown in the conditions that upregulate ECP expression adhered to HeLa (human cervical) cells (Fig. 3). Zhao *et al.* [18] have previously determined that UPEC and APEC strains sharing the same virulence gene profiles were both virulent in chickens and showed the same tendency of iron-acquisition gene expression in a murine model of human UTI. Our data showed that opsonization of bacteria with anti-ECP antibodies resulted in substantial inhibition of bacterial adherence (Fig. 3). These data imply that ECP could be a good antigen candidate to use to protect against ExPEC infection. Our team is in the process of evaluating this antigen to protect chickens against APEC and humans against UPEC, using mouse model for UTI.

### Deletion of *ecpA* and/or *ecpD* Abolishes Synthesis and Expression of ECP in APEC

**Figure 4:**
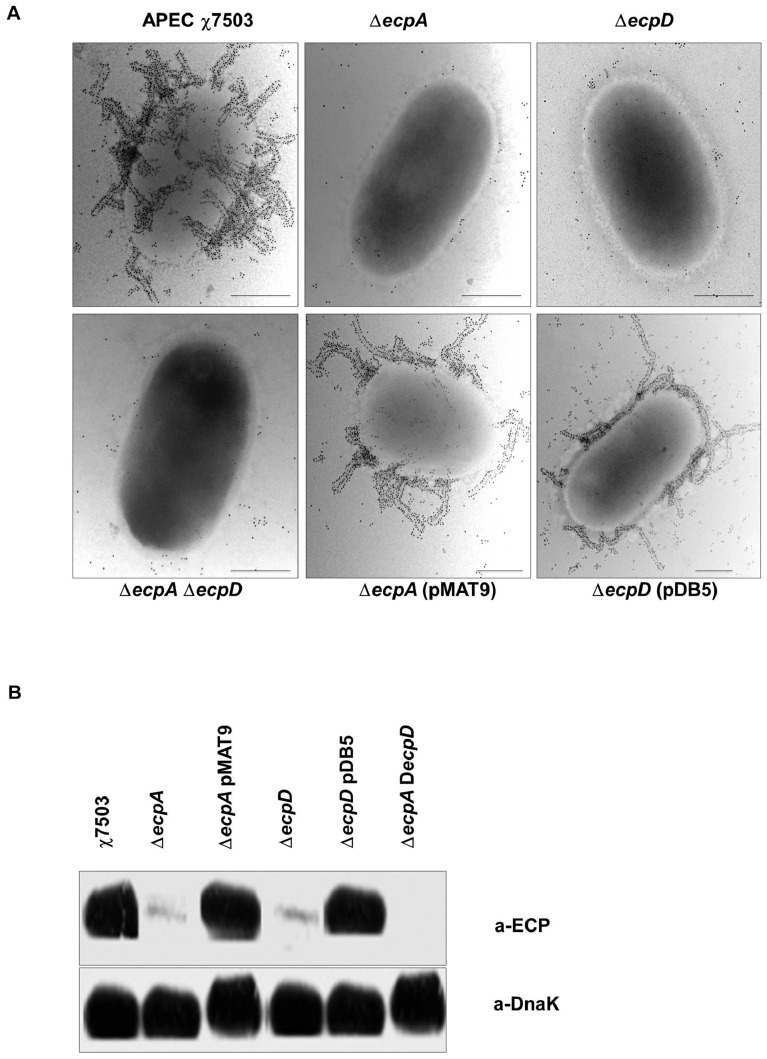
Detection of ECP synthesis and expression on APEC χ7503 strain and its derivatives. ECP expression on the surface of bacteria by immunoelectron microscopy (**A**) and ECP synthesis in total bacterial Western blotting using anti-ECP antibodies (**B**). The ECP synthesis and expression is shown in the wild-type strain. The single and double mutants were deficient in ECP synthesis and expression. The ECP synthesis and expression were restored in the complemented strains. (Scale bars, 500 nm.). Detection of DnaK with anti-DnaK antibody was used to ensure equal amounts of antigen tested.

APEC χ7503 Δ*ecpA,* Δ*ecpD,* and Δ*ecpA*Δ*ecpD* mutant strains were generated (Table 1) to elucidate the role of ECP in various virulence-associated mechanisms in APEC. The two genes *ecpA* and *ecpD* encode for the major pilin EcpA and the polymerized tip adhesin EcpD of the ECP fimbriae, respectively [6]. Similar to studies on human *E. coli*, we have shown that both single mutants and the double mutant in an APEC strain were deficient in ECP synthesis and expression as analyzed by TEM and immunoblotting (Fig. 4). It was obvious that deletion of *ecpA* would affect ECP synthesis; however, the absence of ECP expression in the *ecpD* mutant was surprising and previously explained by the fact that EcpD is required for the stability of EcpA, whereas the absence of EcpA does not affect the expression of EcpD [6]. Complementation of the mutant strains Δ*ecpA* and Δ*ecpD* with the plasmids pMAT9 and pDB5 respectively (Fig. 4), has fully recovered the expression of ECP in the strains. Since deletion of *ecpD* abolishes both EcpD and EcpA expression [6], only the mutant *ecpD* complemented with pDB5 was included in the *in vitro* and *in vivo* assays below.

**Table 1:** *E. coli* strains and plasmids used in this study.

Strains	Characteristics/genotype	Parent	Reference/source
χ7122	APEC O78:K80:H9, *gyrA* Nal^R^ Str^R^		[13]
χ7234	Wild-type APEC O18 isolated from a deceased turkey presenting signs of colibacillosis		Lab. collection
χ7503	Wild-type APEC O1 isolated from a deceased chick presenting signs of colibacillosis		Lab. collection
χ7615	Δ*ecpA::cat*, Cm^R^	χ7503	This study
χ7616	Δ*ecpD::cat*, Cm^R^	χ7503	This study
χ7617	Δ*ecpA::cat* Δ*ecpD::km*, Cm^R^, Km^R^	χ7615	This study
χ7744	χ7615 (Δ*ecpA*) complemented with pMAT9, Cm^R^, Amp^R^	χ7615	This study
χ7745	χ7616 (Δ*ecpD*) complemented with pDB5, Cm^R,^ Am^R^	χ7616	This study
**Plasmids**			
pKD46	Am^R^, λ Red recombinase expression, Temperature sensitive plasmid.		[21]
pKD3	Plasmid containing the Cm cassette		[21]
pKD4	Plasmid containing the Km cassette		[21]
pMAT9	*ecpAB* in pSE380, Am^R^	pSE380	[4]
pDB5	*ecpD* in pBR322, Am^R^	pBR322	[6]

### Diversity of ECP-associated Virulence Phenotypes in APEC χ7503

**Figure 5:**
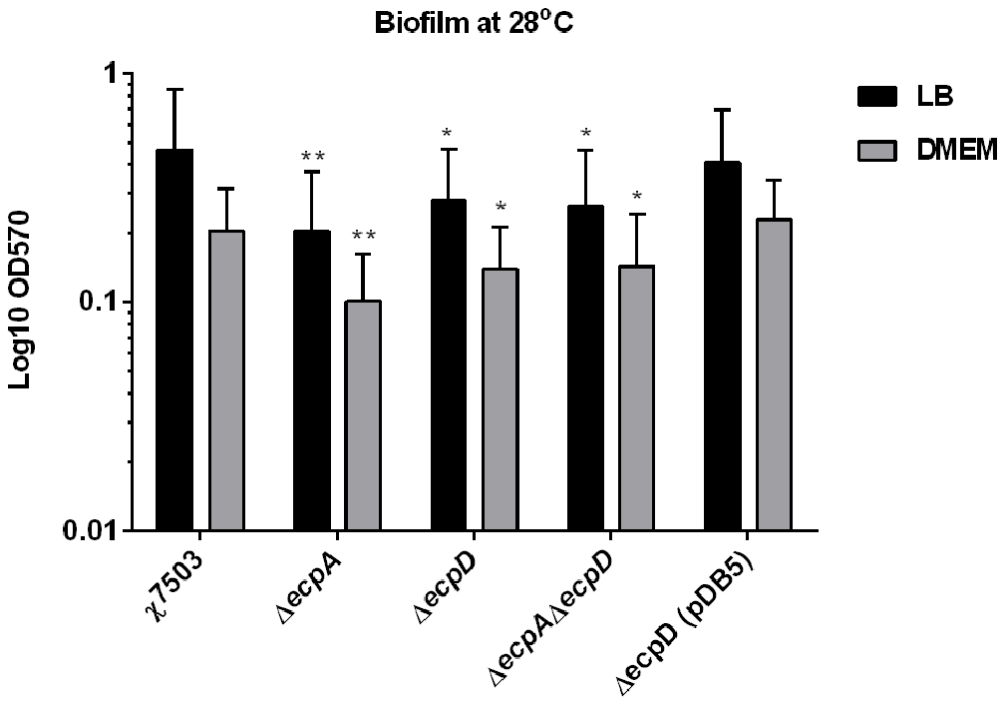
Biofilm formation by APEC χ7503 and its derivatives. Bacteria were grown in either LB or DMEM media at 28°C for 48 hours. Biofilm-associated cells were visualized by crystal violet staining. The data represent means and standard deviations of three independent experiments. Asterisks show significant difference versus the wild-type strain (*, *P*<0.05; **, *P*<0.005).

Biofilm formation provides multiple advantages to bacteria, as it is an important determinant in the pathogenicity of ExPEC, increases survivability of bacteria in the environment outside of the host, and provides an environment for genetic material exchange [19]. Resistance of biofilm-forming bacteria to antimicrobial drugs and detergents complicates the elimination of theses bacteria in medical and industrial settings. A set of gene expressions in *E. coli* facilitate biofilm formation at its different stages including initiation, attachment and maturation. A previous study has shown that ECP was involved in the early stage of biofilm development in NMEC at 20°C and ECP production was detected in biofilm-attached bacteria [8]. Herein, the deletion of *ecpA* and/or *ecpD* in the APEC strain χ7503 has decreased biofilm production in the mutant strains when grown at 28°C in LB or DMEM, and the difference compared to the wild-type was statistically significant (Fig. 5), which confirms that ECP plays a role in biofilm formation in APEC.

**Figure 6:**
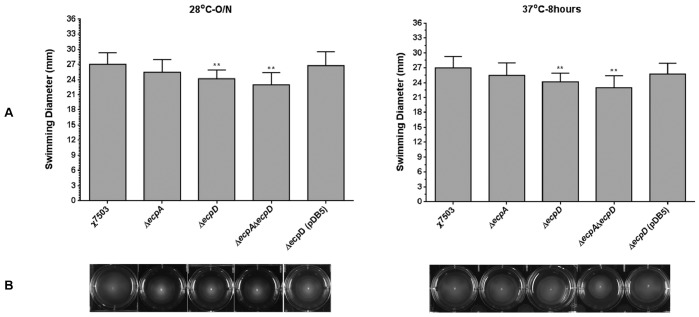
Swimming ability of bacteria in semi-solid LB medium. χ7503 and its *ecp*-derivatives were compared for their swimming ability in semi-solid LB agar at both 28°C and 37°C. The data represent means and standard deviations of three independent experiments. Asterisks show significant difference versus the wild-type strain (*, *P*<0.05).

Lehti *et al.* [8] have shown that overexpression of *matA* (*ecpR*) in NMEC IHE 3034 abolishes the motility of the strain by decreasing the expression of the flagella operon [8]; and the inactivation of *matA* had only a minor effect on flagellation. No studies have been undertaken to evaluate the role of the ECP fimbriae in the motility of bacteria yet. In our present study, we compared the APEC strain χ7503 and its derivative Δ*ecpA*, Δ*ecpD*, and Δ*ecpA*Δ*ecpD* mutants for their motility in a swimming assay on semi-solid agar plates (Fig. 6) and determined that although deletion of *ecpA* in χ7503 had little effect on motility of bacteria, the absence of *ecpD* in both single and double mutants significantly decreased the motility of bacteria compared to their wild-type at both 28°C and 37°C. Complementation of the *ecpD* mutant with the plasmid pDB5 containing the *ecpD* gene has restored the motility of the bacteria to the level of the wild-type (Fig. 6). The mechanism by which ECP is involved in the motility is unclear and has to be elucidated in the future.

### Role of ECP *in vivo*

**Figure 7:**
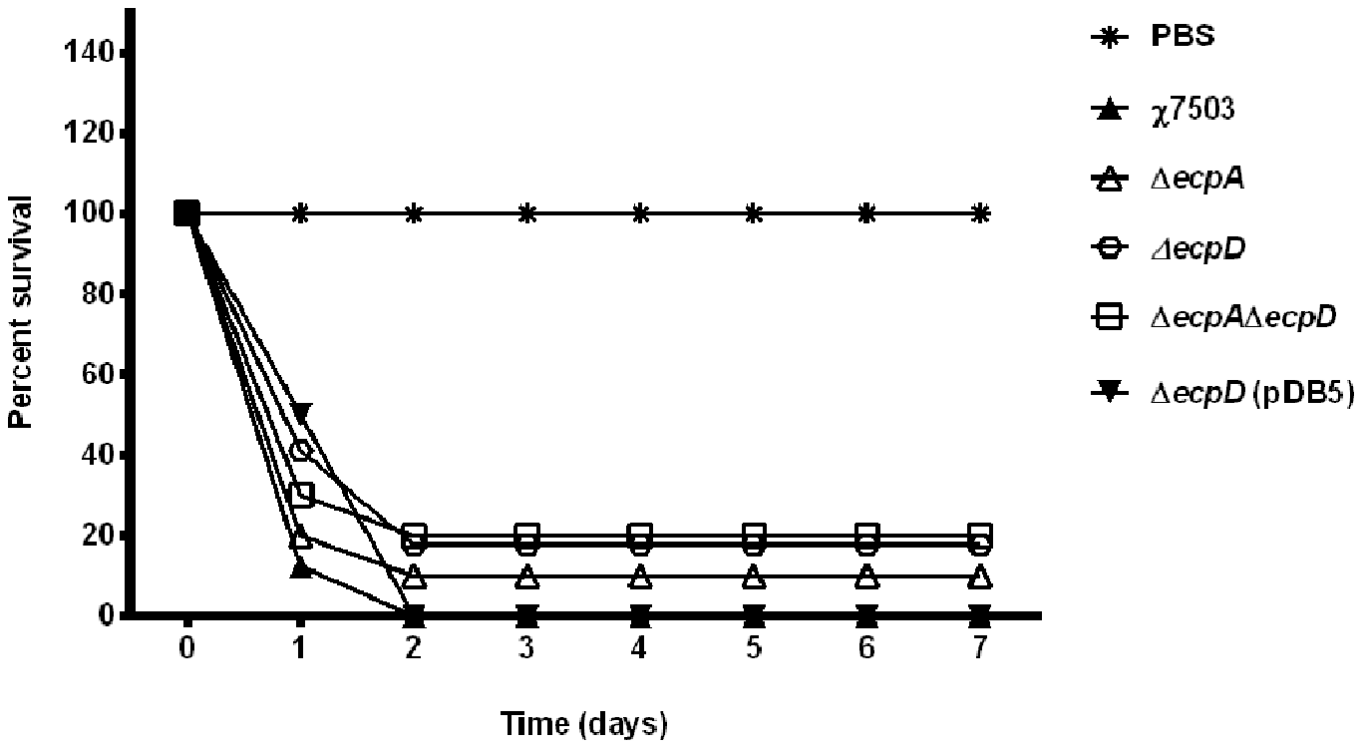
Pathogenicity of APEC χ7503 and its *ecp*-derivative strains in 1-day-old chicks. The survival percentages were evaluated for groups of chicks inoculated subcutaneously at 7 days after inoculation with either wild-type strain χ7503 or its *ecp*-derivative strains. A group of chicks inoculated with PBS was used as a control.

Available studies that assessed the role of ECP in virulence-associated phenotypes of bacteria were undertaken *in vitro* only [4]–[11], [15], [17]. The extent to which the ECP-associated virulence is expressed *in vivo* is speculative. In the present study, evaluation of wild-type APEC χ7503 and its derivative *ecp* mutants in day-old chicks has shown that the wild-type APEC strain χ7503 killed 87.5% of infected chicks at 24 and 100% at 48 hours post-infection (Fig. 7). The *ecpA* mutant killed 80% and 90% at respective times and 10% survived until the end of the experiment; the *ecpD* mutant killed 58.82% and 82.35% and 17.65% survived until the end of the experiment (Fig. 7); the double mutant *ecpA* and *ecpD* killed 70% and 80% respectively and 20% survived until the end of the experiment; and the complemented *ecpD* strain killed 50% and 100% respectively (Fig. 7).

**Figure 8:**
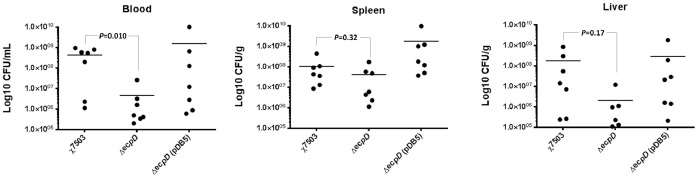
Colonization ability of χ7503 and its *ecp*-derivative strains in 1-day-old chicks. Abilities of strains to cause systemic infection and invade internal organs of chicks were evaluated at 12 hours post-subcutaneous challenge in day-old chicks. Statistically significant differences compared with the wild-type strain are indicated.

Additionally, to evaluate if ECP has a role in colonization during the infection process, day-old chicks were subcutaneously challenged with wild-type, Δ*ecpD* mutant, and its complemented strain, respectively. Data of recovery of bacteria from blood and internal organs (spleen and liver) of infected animals at 12 hours post inoculation, has shown that compared to the wild-type, the Δ*ecpD* mutant colonized the infected animals slowly and had lower mean bacterial populations in blood and internal organs (spleen and liver) and the difference (two logs reduction) was significant (*P<00.5*) in the blood (Fig. 8). Complementation of the mutant strain restored the colonization ability of the strain to the level of the wild-type or even slightly higher in the spleen (Fig. 8).

Although deletion of *ecp* has not drastically attenuated APEC strain χ7503, as tested in the lethality of day-old chick model, this is probably due to ability of bacteria to use alternative virulence factors; its absence has however decreased the colonization ability of the bacteria, especially in blood. To our knowledge, this is the first report on the role of ECP in virulence *in vivo* and we show for the first time a correlation between deletion of *ecp* and the decrease of virulence of APEC in chicks.

In the future studies, *ecp* mutants should be tested in other animal models of avian colibacillosis, such as subcutaneous injection and air sac inoculation [20] to determine their role in specific diseases, including cellulitis and systemic infection respectively.

## Conclusion

We have shown for the first time that *ecpA* is prevalent among APEC isolated from diseased chickens. Our data showed that ECP expression is regulated differently in biofilm and in contact with HeLa cells in *ecpA^+^* APEC strains and is neither serogroup nor phylogenetic group related. Deletion of *ecp* genes in an APEC strain has decreased its biofilm production and swimming ability *in vitro* and has slightly decreased its virulence in day-old chicks and decreased the colonization ability of the strain, especially in bloodstream. Similar to human pathogenic *E. coli*, ECP in APEC is involved in diverse phenotype-associated virulence/fitness and should be considered as a potential common antigen to use against pathogenic *E. coli* infections in both humans and animals.

## Materials and Methods

### Ethics Statement

Infection of chickens was performed in accordance with protocols approved by the Arizona State University (ASU) Institutional Animal Care and Use Committee (IACUC) in dedicated facilities at the Biodesign Institute, ASU (ASU IACUC Protocol number 1168R).

During the experiment, chicks were regularly monitored four times a day by our team and the facility animal caretakers, and further inspected by an ASU veterinarian. Chickens were monitored for death as an endpoint. However, any moribund chicks (very sick and no motility, obviously in pain and showing signs of severe and enduring distress) were euthanized by CO_2_ asphyxiation to minimize suffering of these animals.

### Bacterial Strains, Plasmids and Growth Conditions

The list and characteristics of *Escherichia coli* strains and plasmids used in this study are in Table 1. A collection of 166 *E. coli* strains isolated from chickens and turkeys presenting signs of colibacillosis [12] [This study], kindly provided by Dr. John Fairbrother (University of Montreal), and the APEC strain χ7122 [13] were used to study the distribution of the *ecpA* gene among APEC using PCR. The APEC strain χ7503 from the serogroup O1 contains four plasmids (∼114 kb, ∼105 kb, ∼76.5 kb, and ∼55 kb); it was PCR-tested positive for six ColV plasmid-associated genes (*iss, cvi, ompT, iroN, iutA,* and *tsh*) that play a major role in virulence of APEC [12] and for colicin and siderophore production using methods previously described [19]. Unless otherwise stated, bacteria were routinely grown in Luria Bertani (LB) broth or on MacConkey agar supplemented with 0.1% glucose and 1% lactose respectively at 37°C. Strains were stored as stock cultures at −80°C in peptone-glycerol medium. Antibiotic susceptibility of strains was tested on LB-agar plates with and without antibiotics and on Muller Hinton agar using antibiotic disks. Antibiotics were added, as required, at the following concentrations (µg/ml): kanamycin (30); chloramphenicol (50); and ampicillin (100).

### PCR Amplification, Sequencing and Computational Analysis

PCR amplification of DNA was performed using Gotaq® DNA Polymerase (Promega). PCR reaction products were resolved on a 1% agarose gel and visualized with Syber-green staining with UV or blue light source. Detection of *ecpA* in 167 APEC isolates and ColV plasmid-associated genes (*iss, cvi, ompT, iroN, iutA, tsh*) in χ7503 was performed by PCR amplification using primers specified in Table 2 and as previously described [12]. For comparison purposes, the sequence of the operon *ecpRABCDE* was derived from the whole genomic DNA of APEC χ7122 (NZ_HE962388.1), CFT073 (NC_004431.1), and E2348/69 (FM180568.1). BLAST programs (http://www.ncbi.nlm.nih.gov) were used to carefully review, confirm the annotation of every gene and compare between sequences.

**Table 2:** Primers used in this study.

Primer	Sequence	Target gene/purpose	Reference
***ecpA*** **-L**	GTAACGGTGTTTACCGGCAT	*ecpA* (screening)	This study
***ecpA*** **-R**	GATCATCACGGTATCGCCAG	*ecpA* (screening)	This study
**G60**	GTTCTGGCAATAGCTCTGGTAACGGTGTTTACCGGCGTGTAGGCTGGAGCTGCTTC	*ecpA* (mutagenesis)	[5]
**G61**	TTAACTGGTCCAGGTCGCGTCGAACTGTACGCTAACCATATGAATATCCTCCTTAG	*ecpA* (mutagenesis)	[5]
**G90**	AACAGCAATATTAGGGGCGTG	*ecpA* (screening mutant)	[5]
**G91**	GGATAACAGCAGAGCGAGAAG	*ecpA* (screening mutant)	[5]
***ecpD*** **-cat/km-L**	GTGCCGCCAGCATACAGACCGCTGTCAGCAGGGCCGTGTAGGCTGGAGCTGCTTC	*ecpD* (mutagenesis)	This study
***ecpD*** **-cat/km-R**	CATCGTGGGCGGCGGTGACGCAGACAGGAGAAGAGACATATGAATATCCTCCTTAG	*ecpD* (mutagenesis)	This study
***ecpD*** **-V-F**	ATAGCACTGATGGCAATACG	*ecpD* (screening mutant)	This study
***ecpD*** **-V-R**	GTGGCACTGGAACTCAACCA	*ecpD* (screening mutant)	This study

For sequencing the *ecp* upstream region that includes the *ecp* promoter from selected APEC strains, DNA templates were generated by PCR using primers specified in Table 2. The PCR products were purified from the agarose gel by using Qiaquick Gel Extraction Kit (Qiagen) and sequenced by the DNA laboratory Sequencing Core at Arizona State University (https://sols.asu.edu/about-us/labs/dna). The sequence data have been deposited in the GenBank database under Accession numbers: χ7499 (KF366455); χ7237 (KF366456); χ7244 (KF366457); χ7503 (KF366458); χ7507 (KF366459); χ7508 (KF366460); χ7514 (KF366461); χ7516 (KF366462); χ7258 (KF366463); χ7520 (KF366464); χ7233 (KF366465); χ7521 (KF366466); χ7234 (KF366467); χ7254 (KF366468); χ7523 (KF366469); χ7528 (KF366470); χ7535 (KF366471); χ7537 (KF366472); χ7543 (KF366473); χ7545 (KF366474); χ7554 (KF366475); χ7555 (KF366476); χ7560 (KF366477); 16269 (KF907797); 16331 (KF907798); 16423 (KF907799); 16924 (KF907800); 16998 (KF907801); 17002 (KF907802); 17162 (KF907803); 17270 (KF907804); 17310 (KF907805); 17328 (KF907806); 17343 (KF907807); 17355 (KF907808); 17504 (KF907809); 17669 (KF907810); 17713 (KF907811); 17840 (KF907812); 17914 (KF907813); and 19268 (KF907814).

A phylogenetic tree was generated by comparing the nucleotide levels of these sequences and the sequences of APEC available on the public database with those of related sequences of other bacteria obtained from the GenBank database by neighbor-joining (1,000 replicates) using Molecular Evolutionary Genetics Analysis software version 4.0 (MEGA4) (http://megasoftware.net/). Bootstrap values are indicated at branch positions. GenBank accession numbers of nucleotide sequences of APEC and other bacteria used in this study are: χ7122 (NZ_HE962388.1), CFT073 (NC_004431.1), APEC O1 (NC_008563.1); BEN374 (JN377377); BEN79 (JN377376); CB9615 (CP001846); SMS-3-5 (CP000970); E2348/69 (FM180568.1); DH1 (CP001637); UMN026 (CU928163.1); 789 (JN377380); TW14359 (CP001368); IMT2125 (NZ_HE964769); E24377A (CP000800); APEC O78 (NC_020163.1); BL21(DE3) (NC_012892.2); 55989 (CU928145.2); 11368 (AP010953); *Shigella boydii* Sb227 (NR_076357.1); *K. pneumoniae* Ec18 (NZ_HF536482.1).

### Construction of Mutants

Genes *ecpA* and/or *ecpD* were deleted in one of the APEC strains tested, named χ7503 (Table 1), using λ Red-recombineering technology [21], a one-step gene inactivation method using primers listed in Table 2. The strain χ7503 was selected because it tested *ecpA^+^* and ECP+, the strain was from the serogroup O1 (Fig. 2), one of the most prevalent serotypes among APEC isolates [1], and tested positive for most genes associated with APEC as determined above. Insertions and deletions in generated mutant strains were verified by PCR using the primers in Table 2. The LPS profile of strains was evaluated by sodium dodecyl sulfate-polyacrylamide gel electrophoresis (SDS-PAGE) and visualized by silver staining [22], to eliminate rough variants if they arise. Strains were verified for their similar growth and antibiotic sensitivity.

### Bacterial Interaction with HeLa Cells

HeLa cells at 70–80% confluence were cultivated in 24-well tissue culture plates containing glass coverslips and DMEM supplemented with 10% bovine fetal serum (Gibco Invitrogen, USA) and 1% antibiotics (Gibco Invitrogen, USA). After one wash with phosphate-buffered saline (PBS) pH 7.4, 1.0 mL of fresh medium (DMEM supplemented with 2% fetal bovine serum) was added to the cell monolayers [9]. APEC strains were grown overnight in DMEM broth without shaking at 28°C. Bacteria were diluted 1∶100 in the medium contained in the microplates. After an incubation time of 6 hours at 37°C, cells were washed with phosphate buffered saline (PBS) to remove non-adherent bacteria. Cells were fixed with 2% formaldehyde and were either stained using a Giemsa staining kit or used for immunofluorescence microscopy as described below [5].

To examine the ability of anti-ECP antibodies to inhibit adherence, the bacterial inoculum was pre-incubated for 30 min with 1∶10, 1∶50, and 1∶100 dilutions of the anti-ECP before addition to the cells.

### Biofilm Assay

To measure biofilm formation in bacteria, assays were performed in 96-well polystyrene microtiter plates (Becton Dickinson, Franklin Lakes, NJ). An O/N standing LB culture of the strains was diluted 1∶100 in fresh media of either LB or DMEM low glucose with 0.5% Mannose. Aliquots of 200 µL for each dilution were dispensed per well into a microtiter plate. Each strain was tested in multiples of twelve, with wells containing sterile medium used as negative controls. After incubation for 48 h at 28°C, biofilms were quantified using Crystal Violet staining. The absorbance was measured at 570 nm in an absorbance spectrophotometer (SpectraMax M2, Molecular Devices). All tests were carried out at least three times, and the results were averaged.

Biofilm-associated bacteria for evaluation of ECP production assay was performed in 24-well plates (Nunc) with glass coverslips. A 20-µl aliquot of a standing O/N culture of bacteria grown in LB broth was added to the wells containing 500 µl DMEM low glucose containing 0.5% mannose and incubated at 28°C for 48 h. Wells were washed three times with PBS to remove unbound bacteria and the biofilms were fixed with 2% formalin and processed for IFM or TEM as described below.

### Detection of ECP Expression by Immunoblotting

Overnight bacterial cultures obtained from DMEM were adjusted to an absorbance of 1.1 at OD_600_. Equal numbers of bacteria were used to prepare whole-cell extracts after treatment with acidified water (pH 1.2), boiling for 5 min, addition of SDS-PAGE sample buffer and neutralization with 1 N NaOH as previously described [5]. The samples were electrophoresed in 16% polyacrylamaide gels under denaturing SDS-PAGE conditions. Detection of DnaK with anti-DnaK antiserum (Sigma Aldrich) served as a control for equal amounts of protein loaded onto the gels. The proteins were electroblotted onto PVDF membranes, blocked with 1% dry milk, and the immobilized proteins were bound with primary antibodies against ECP, followed by incubation with goat anti-rabbit IgG conjugated to peroxidase (Sigma Aldrich). The substrate used was a chemo-luminescent reagent (Amersham).

### Detection of surface expression of ECP

Surface expression of ECP in biofilm- or cell-associated bacteria was visualized via immunofluorescence and TEM using Rabbit anti-EcpA antibodies [5]. Immunofluoresence was accomplished using Alexa Fluor conjugated anti-Rabbit antibodies (Green) at a concentration of 1∶7,500 in PBS. Bacterial cells were stained with Propidium Iodide. ECP was visualized under TEM using Goat-anti-Rabbit IgG with 10 nm colloidal gold (MP Biomedicals) at a concentration of 1∶250.

### Ultrastructural Analysis of ECP Expression by Electron Microscopy

DMEM bacterial cultures were spotted onto 300-mesh carbon-Formvar copper grids, negatively stained with 10 µl of 1% phosphotungstic acid (pH 7.4) for 5 min, and analyzed for the presence of pili by transmission electron microscopy (TEM). Immuno-EM studies were performed to confirm the presence of ECP by incubating the bacteria for 1 h with rabbit anti-ECP antibody (diluted 1∶10) in PBS containing 10% BSA and 1 h-incubation with goat anti-rabbit IgG conjugated to 10-nm gold particles diluted 1∶10 (BB International) as previously described [23].

### Motility Assays

Isolated colonies of each strain from an O/N fresh LB plates were inoculated with sterile toothpicks on swimming plates (1.0% Difco Bactotryptone, 0.5% Difco Yeast Extract, 0.5% NaCl, and 0.3% Difco Agar) prepared the same day and dried for 6 hours before inoculation. Plates were incubated either O/N at room temperature or 6 hours at 37°C. Swimming halo diameters were measured. At least six colonies from each strain were tested, and the test was repeated at least twice.

### Evaluation of the Virulence of the Strains *in vivo*

Specific-pathogen-free fertile White Leghorn chicken eggs were obtained from Charles River Labs (Wilmington, MA) and hatched at the animal facilities of the Biodesign Institute. During the study chickens were housed in isolators equipped with HEPA filters in the BSL2 facilities.

Lethality for 1-day-old chicks was assessed by subcutaneously inoculating 5 groups of 10 1-day-old chicks with 0.1 ml of either PBS or an overnight broth culture of strains (about 10^8^ CFU) [24]. Death/survival was recorded for 7 days after inoculation.

The difference in the abilities of strains to disseminate in the bloodstream and internal organs of chicks was also determined. Briefly, 3 groups of 7 day-old chicks were subcutaneously inoculated with 10^8^ CFU of either the wild-type, Δ*ecpD* mutant, or its complemented strain respectively. Birds were observed every two hours and euthanized at 12 h post-infection by CO_2_ asphyxiation and then necropsied. Blood was collected in heparinized syringes and organs (spleen and liver) were aseptically removed and homogenized in PBS, the presence and number of bacteria were determined by plating serial dilutions of samples on MacConkey agar plates.

### Statistical Analysis

Data were analyzed by one-way analysis of variance (ANOVA), followed by Bonferroni’s multiple-comparison test (GraphPad Prism software, version 6.01). Differences between average values were also tested for significance by performing an unpaired, two-sided Student *t* test. The levels of significance (*P* values) are reported and values ≤0.05 were taken to be significant.

## Supporting Information

Figure S1 **Schematic of the Genetic organization of the** ***ecp*** **operon of APEC χ7122.** Arrows represent genes of the *ecp* operon. The numbers inside of the arrows represent the size of the genes in base pairs (bp).
(TIF)

Table S1 Percent sequence ***identity*** and ***positive*** substitutions of ECP protein sequences of χ7122 compared to two ECP+ strain (CFT073) [6] and E2348/69 [5], [14] and one ECP- strain (APEC-O1) (NC_008563.1) (this study).
(DOC)

## References

[1] Barnes HJ, Vaillancourt J, Gross WB (2003) Colibacillosis. In: Saif YM, editor. Diseases of poultry: Iowa State University Press, Ames, Iowa. 631–652.

[2] Dziva F, Stevens MP (2008) Colibacillosis in poultry: unravelling the molecular basis of virulence of avian pathogenic *Escherichia coli* in their natural hosts. Avian Pathol 37: 355–366. 18622850 10.1080/03079450802216652

[3] Moulin-Schouleur M, Reperant M, Laurent S, Bree A, Mignon-Grasteau S, et al (2007) Extraintestinal pathogenic *Escherichia coli* strains of avian and human origin: link between phylogenetic relationships and common virulence patterns. J Clin Microbiol 45: 3366–3376. 17652485 10.1128/JCM.00037-07 PMC2045314

[4] Pouttu R, Westerlund-Wikstrom B, Lang H, Alsti K, Virkola R, et al (2001) *matB*, a common fimbrillin gene of *Escherichia coli*, expressed in a genetically conserved, virulent clonal group. J Bacteriol 183: 4727–4736. 11466275 10.1128/JB.183.16.4727-4736.2001 PMC99526

[5] Rendon MA, Saldana Z, Erdem AL, Monteiro-Neto V, Vazquez A, et al (2007) Commensal and pathogenic *Escherichia coli* use a common pilus adherence factor for epithelial cell colonization. Proc Natl Acad Sci U S A 104: 10637–10642. 17563352 10.1073/pnas.0704104104 PMC1890562

[6] Garnett JA, Martinez-Santos VI, Saldana Z, Pape T, Hawthorne W, et al (2012) Structural insights into the biogenesis and biofilm formation by the *Escherichia coli* common pilus. Proc Natl Acad Sci U S A 109: 3950–3955. 22355107 10.1073/pnas.1106733109 PMC3309717

[7] Avelino F, Saldana Z, Islam S, Monteiro-Neto V, Dall’Agnol M, et al (2010) The majority of enteroaggregative *Escherichia coli* strains produce the *E. coli* common pilus when adhering to cultured epithelial cells. Int J Med Microbiol 300: 440–448. 20452276 10.1016/j.ijmm.2010.02.002

[8] Lehti TA, Bauchart P, Heikkinen J, Hacker J, Korhonen TK, et al (2010) Mat fimbriae promote biofilm formation by meningitis-associated *Escherichia coli* . Microbiology 156: 2408–2417. 20522494 10.1099/mic.0.039610-0

[9] Saldana Z, Erdem AL, Schuller S, Okeke IN, Lucas M, et al (2009) The *Escherichia coli* common pilus and the bundle-forming pilus act in concert during the formation of localized adherence by enteropathogenic *E. coli* . J Bacteriol 191: 3451–3461. 19218393 10.1128/JB.01539-08 PMC2681888

[10] Hernandes RT, Velsko I, Sampaio SC, Elias WP, Robins-Browne RM, et al (2011) Fimbrial adhesins produced by atypical enteropathogenic *Escherichia coli* strains. Appl Environ Microbiol 77: 8391–8399. 21926222 10.1128/AEM.05376-11 PMC3233042

[11] Blackburn D, Husband A, Saldana Z, Nada RA, Klena J, et al (2009) Distribution of the *Escherichia coli* common pilus among diverse strains of human enterotoxigenic *E. coli* . J Clin Microbiol 47: 1781–1784. 19357209 10.1128/JCM.00260-09 PMC2691072

[12] Mellata M, Touchman JW, Curtiss III R (2009) Full sequence and comparative analysis of the plasmid pAPEC-1 of avian pathogenic *E. coli* chi7122 (O78:K80:H9). PLoS One 4: e4232. 19156210 10.1371/journal.pone.0004232 PMC2626276

[13] Brown PK, Curtiss 3rd R (1996) Unique chromosomal regions associated with virulence of an avian pathogenic *Escherichia coli* strain. Proc Natl Acad Sci U S A 93: 11149–11154. 8855324 10.1073/pnas.93.20.11149 PMC38299

[14] Lehti TA, Bauchart P, Kukkonen M, Dobrindt U, Korhonen TK, et al (2013) Phylogenetic group-associated differences in regulation of the common colonization factor Mat fimbria in *Escherichia coli* . Mol Microbiol 87: 1200–1222. 23347101 10.1111/mmi.12161

[15] Lehti TA, Bauchart P, Dobrindt U, Korhonen TK, Westerlund-Wikstrom B (2012) The fimbriae activator MatA switches off motility in *Escherichia coli* by repression of the flagellar master operon *flhDC* . Microbiology 158: 1444–1455. 22422754 10.1099/mic.0.056499-0

[16] Manges AR, Johnson JR (2012) Food-borne origins of *Escherichia coli* causing extraintestinal infections. Clin Infect Dis 55: 712–719. 22615330 10.1093/cid/cis502

[17] Scaletsky IC, Aranda KR, Souza TB, Silva NP (2010) Adherence factors in atypical enteropathogenic *Escherichia coli* strains expressing the localized adherence-like pattern in HEp-2 cells. J Clin Microbiol 48: 302–306. 19864474 10.1128/JCM.01980-09 PMC2812252

[18] Zhao L, Gao S, Huan H, Xu X, Zhu X, et al (2009) Comparison of virulence factors and expression of specific genes between uropathogenic *Escherichia coli* and avian pathogenic *E. coli* in a murine urinary tract infection model and a chicken challenge model. Microbiology 155: 1634–1644. 19372154 10.1099/mic.0.024869-0

[19] Mellata M, Maddux JT, Nam T, Thomson N, Hauser H, et al (2012) New insights into the bacterial fitness-associated mechanisms revealed by the characterization of large plasmids of an avian pathogenic *E. coli* . PLoS One 7: e29481. 22238616 10.1371/journal.pone.0029481 PMC3251573

[20] Dziva F (2010) Deciphering the infection biology of avian pathogenic *Escherichia coli:* role of experimental infection models. In: Mendez-Vilas A, editor. Current Research, Technology and Education Topics in Applied Microbiology and Microbial Biotechnology. Spain: Formatex Research Center. 746–753.

[21] Datsenko KA, Wanner BL (2000) One-step inactivation of chromosomal genes in *Escherichia coli* K-12 using PCR products. Proc Natl Acad Sci U S A 97: 6640–6645. 10829079 10.1073/pnas.120163297 PMC18686

[22] Hitchcock PJ, Brown TM (1983) Morphological heterogeneity among *Salmonella* lipopolysaccharide chemotypes in silver-stained polyacrylamide gels. J Bacteriol 154: 269–277. 6187729 10.1128/jb.154.1.269-277.1983 PMC217456

[23] Giron JA, Torres AG, Freer E, Kaper JB (2002) The flagella of enteropathogenic *Escherichia coli* mediate adherence to epithelial cells. Mol Microbiol 44: 361–379. 11972776 10.1046/j.1365-2958.2002.02899.x

[24] Mellata M, Ameiss K, Mo H, Curtiss 3rd R (2010) Characterization of the contribution to virulence of three large plasmids of avian pathogenic *Escherichia coli* chi7122 (O78:K80:H9). Infect Immun 78: 1528–1541. 20086082 10.1128/IAI.00981-09 PMC2849417

