# Supplementary figures and images for: Evaluation of the Prevalence and Production of Escherichia coli Common Pilus among Avian Pathogenic E. coli and Its Role in Virulence
Source: PLoS One. 2014 Jan 23;9(1):e86565. doi: 10.1371/journal.pone.0086565 (PMC3900561; doi:10.1371/journal.pone.0086565)

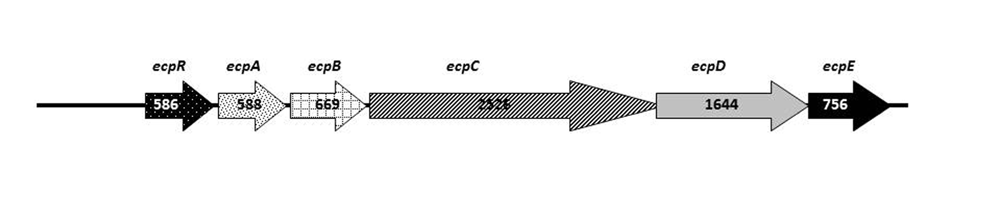

Supplement: Figure S1 — Schematic of the Genetic organization of the ecp operon of APEC χ7122. Arrows represent genes of the ecp operon. The numbers inside of the arrows represent the size of the genes in base pairs (bp). (TIF) [file pone.0086565.s001.tif]
