# Supplementary material for: Evaluation of the Prevalence and Production of Escherichia coli Common Pilus among Avian Pathogenic E. coli and Its Role in Virulence
Source: PLoS One. 2014 Jan 23;9(1):e86565. doi: 10.1371/journal.pone.0086565 (PMC3900561; doi:10.1371/journal.pone.0086565)
Supplement: Table S1 — Percent sequence identity and positive substitutions of ECP protein sequences of χ7122 compared to two ECP+ strain (CFT073) [6] and E2348/69 [5], [14] and one ECP- strain (APEC-O1) (NC_008563.1) (this study). (DOC) [file pone.0086565.s002.doc]

**Table 1S**: Percent sequence identity and positive substitutions of ECP protein sequences of 7122 compared to two ECP+ strain (CFT073) [6] and E2348/69 [5,14] and one Ecp- strain (APEC-O1) (this study).

| **Strains compared to** | **Identity/**  **similarity** | **Proteins** | | | | | |
| --- | --- | --- | --- | --- | --- | --- | --- |
|  |  | **EcpR** | **EcpA** | **EcpB** | **EcpC** | **EcpD** | **EcpE** |
| **CFT073 (ECP+)** | **Identity** | 189/195(97%) | 192/195(98%) | 220/222(99%) | 829/841(99%) | 542/547(99%) | 246/251(98%) |
|  | **Positive** | 193/195(98%) | 193/195(98%) | 222/222(100%) | 835/841(99%) | 542/547(99%) | 249/251(99%) |
|  | **Gaps** | 0/195(0%) | 0/195(0%) | 0/222(0%) | 0/841(0%) | 0/547(0%) | 0/251(0%) |
| **APEC-O1 (ECP-)** | **Identity** | 188/195(96%) | 194/195(99%) | 219/222(99%) | 837/841(99%) | 541/547(99%) | 245/251(98%) |
|  | **Positive** | 193/195(98%) | 195/195(100%) | 220/222(99%) | 839/841(99%) | 541/547(98%) | 249/251(99%) |
|  | **Gaps** | 0/195(0%) | 0/195(0%) | 0/222(0%) | 0/841(0%) | 0/547(0%) | 0/251(0%) |
| **EPEC E23 (ECP+)** | **Identity** | 189/195(97%) | 193/195(99%) | 220/222(99%) | 830/841(99%) | 541/547(99%) | 226/230(98%) |
|  | **Positive** | 193/195(98%) | 194/195(99%) | 222/222(100%) | 836/841(99%) | 541/547(98%) | 228/230(99%) |
|  | **Gaps** | 0/195(0%) | 0/195(0%) | 0/222(0%) | 0/841(0%) | 0/547(0%) | 0/230(0%) |
